# Use of Neoveil or TachoSil to prevent pancreatic fistula following pancreaticoduodenectomy: A retrospective study

**DOI:** 10.1097/MD.0000000000015293

**Published:** 2019-04-26

**Authors:** Hye Eun Kwon, Hyung-Il Seo, Sung Pil Yun

**Affiliations:** a Department of Surgery, Biomedical Research Institute, Pusan National University Hospital, Gudeok-ro, Seo-gu; b Department of Surgery, On Hospital, Busan, Korea.

**Keywords:** pancreas, pancreatic fistula, pancreaticoduodenectomy, pancreaticojejunostomy, PD, POPF, PPPD

## Abstract

The aim of this study was to evaluate the effectiveness of using Neoveil and TachoSil sponges at the pancreaticojejunostomy anastomosis site in reducing the rate and severity of postoperative pancreatic fistula (POPF).

In this study, we retrospectively evaluated data that were prospectively collected on pancreaticoduodenectomy (PD) procedures. Patients were divided into 3 groups: no patch application, Neoveil patch application, and TachoSil patch application. Demographic and surgical data were analyzed.

Around 165 patients with PD were enrolled in this study and were divided into 3 groups. In the standard group (n = 43), no patch was applied, while in the Neoveil and TachoSil groups (n = 84 and n = 38, respectively), the pancreaticojejunostomy anastomosis site was covered with Neoveil and TachoSil patches, respectively. POPF grade B or above occurred in 37.2% (16/43), 14.3% (12/84), and 18.8% (6/38) of patients in the standard, Neoveil and TachoSil groups, respectively, with a significant difference between patients with and without patch application (*P = *.004). On multivariate logistic analysis of predictive factors for POPF, male sex, patch application, and hospital day were found to be the significant independent predictors of POPF grade B or above.

Significant independent predictors of POPF were male sex and patch application. This study demonstrated that the use of Neoveil or TachoSil patches may reduce the incidence of POPF after PD.

## 1 Introduction

Pancreaticoduodenectomy (PD) is the only curative treatment for periampullary malignant or premalignant disease. Despite recent advances in surgical techniques and postoperative care, the morbidity rate remains high (5%–40%). The mortality rate after PD is <5% in high volume centers.^[1–5]^

The most frequent clinically significant complication after PD is postoperative pancreatic fistula (POPF), which lead to secondary complications, such as intra-abdominal abscess, sepsis, and bleeding. Despite attempts at reducing the incidence of POPF, including several methods of pancreaticoenteric anastomosis, fibrin sealants, pancreatic stent insertion, and administration of octreotide, the incidence of POPF after PD has not notably decreased.

The usefulness of using tissue sealants at the anastomosis site is still debated.^[6–8]^ Neoveil is a bioabsorbable soft-tissue reinforcement material that is derived from 100% polyglycolic acid. TachoSil is a topical absorbable fibrin sealant patch that consists of a collagen fleece coated with human fibrinogen and thrombin. The aim of this study was to evaluate the effectiveness of the application of Neoveil and TachoSil patches at the pancreaticojejunostomy anastomosis site in reducing the rate and severity of POPF.

## 2 Methods

Around 165 patients who underwent PD or pylorus-preserving pancreaticoduodenectomy (PPPD) between January 2012 and December 2015 were enrolled in this study. This is a retrospective study of data collected prospectively after PD. This study was conducted by a single surgeon who had previously performed more than 80 PD or PPPD procedures. The clinicopathological data were analyzed retrospectively using our electronic medical database. Factors evaluated included patient sex, age, diagnosis, body mass index (BMI), operative time, estimated blood loss (EBL), surgical procedure, and length of hospital stay. Patients were divided in 3 groups according to time schedule.

The research was conducted by one surgeon who had previously performed more than 50 PD procedures. Patients were categorized into the standard group performed between January and December 2012, the Neoveil group performed between January 2013 and December 2015, and the TachoSil group performed between January and December 2016.

**Figure 1:**
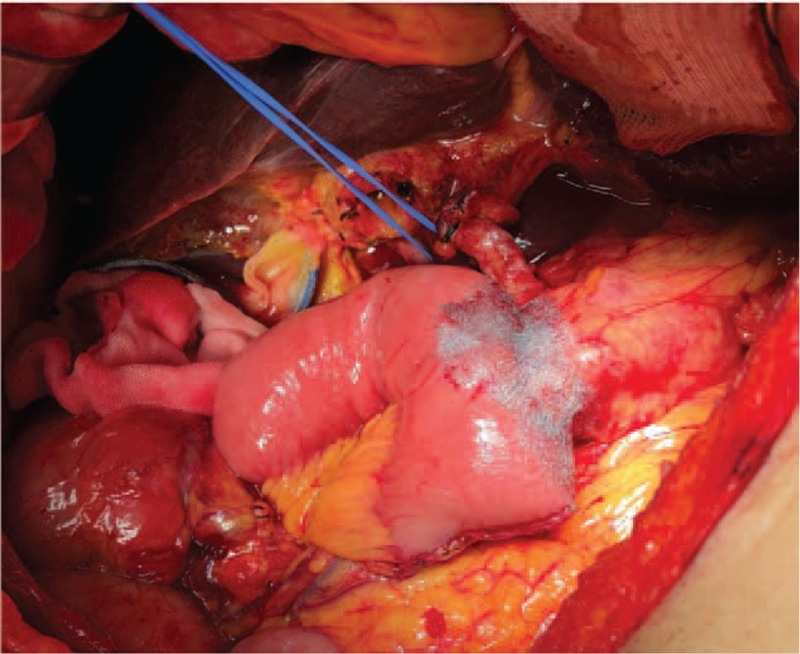
Neoveil patch applied at the pancreaticojejunostomy site.

**Figure 2:**
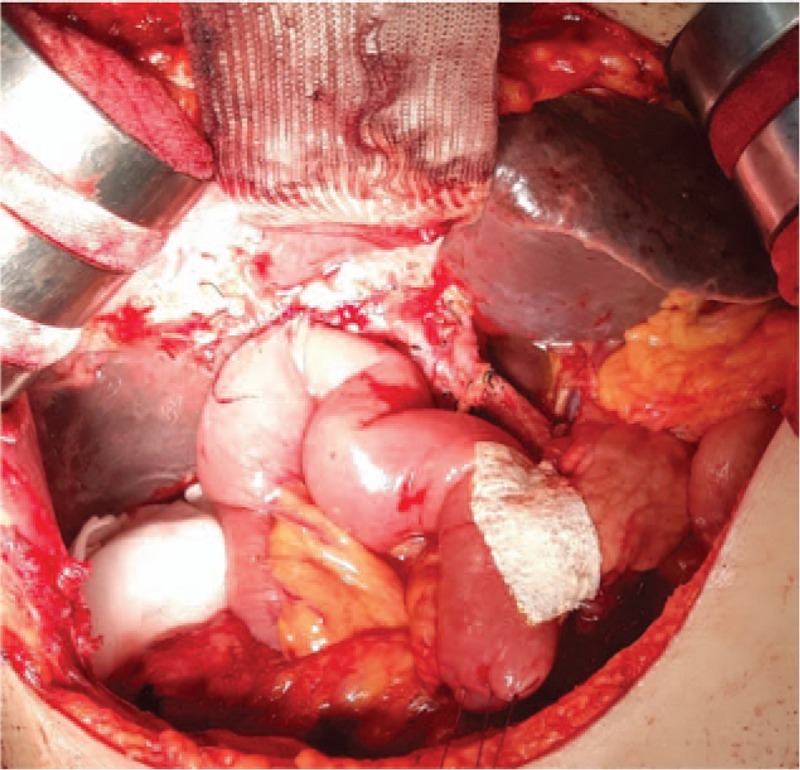
TachoSil patch applied at the pancreaticojejunostomy site.

In the standard group (n = 43), no patch was applied, while in the Neoveil group (n = 84) and TachoSil group (n = 38), the pancreaticojejunostomy anastomosis site was covered with Neoveil (Gunze Co., Ltd, Tokyo, Japan) (Fig. 1) and TachoSil (Nycomed, Pharmaceutical Co. Ltd, Denmark) (Fig. 2), respectively. All pancreaticoenteric anastomoses were performed by double-layered, duct-to-mucosa, end-to-side pancreaticojejunostomy with an internal stent (polyethylene tube). Following complete anastomosis, a closed suction silicon drain (Jackson-Pratt, Baxter Health Care Corp., Deerfield, IL, USA) was placed near the pancreaticojejunostomy site and was retained for at least 7 days postoperatively to prevent intra-abdominal fluid collection and to detect POPF. On the 7th day postoperatively, we checked the amylase concentration in the drainage fluid and performed computed tomography to determine the effect of Neoveil and TachoSil on the incidence of POPF.

POPF was defined according to the International Study Group on Pancreatic Fistula criteria, which defined pancreatic fistula as a measurable volume of drainage fluid with an amylase concentration >3 times the upper limit of normal. Three different grades of POPF are defined according to the patient's postoperative clinical course.^[9]^ We used Clavien-Dindo classification for quality assessment in PD or PPPD.^[10]^

This retrospective study was approved by the institutional review board at Pusan National University Hospital Clinical Trial Center (IRB No. H-1901-007-074).

### 2.1 Statistical analysis

Data distribution was verified by the Shapiro–Wilk test. Categorical variables were compared between the 3 groups using the chi-square or Fisher's exact test. The continuous variables were compared using Student *t* test or Mann–Whitney *U* test, where appropriate. A logistic regression model was used for univariate and multivariate analysis. *P* values <.05 were considered statistically significant. All statistical analyses were performed using SPSS version 23.0 for Windows (IBM Corp., Armonk, NY).

## 3 Results

Out of 165 patients, 105 underwent PPPD and 60 underwent PD. There were no differences in sex, age, pathologic result, operation type, BMI, operation time, EBL, or length of hospital stay in each group (Table 1).

**Table 1:**
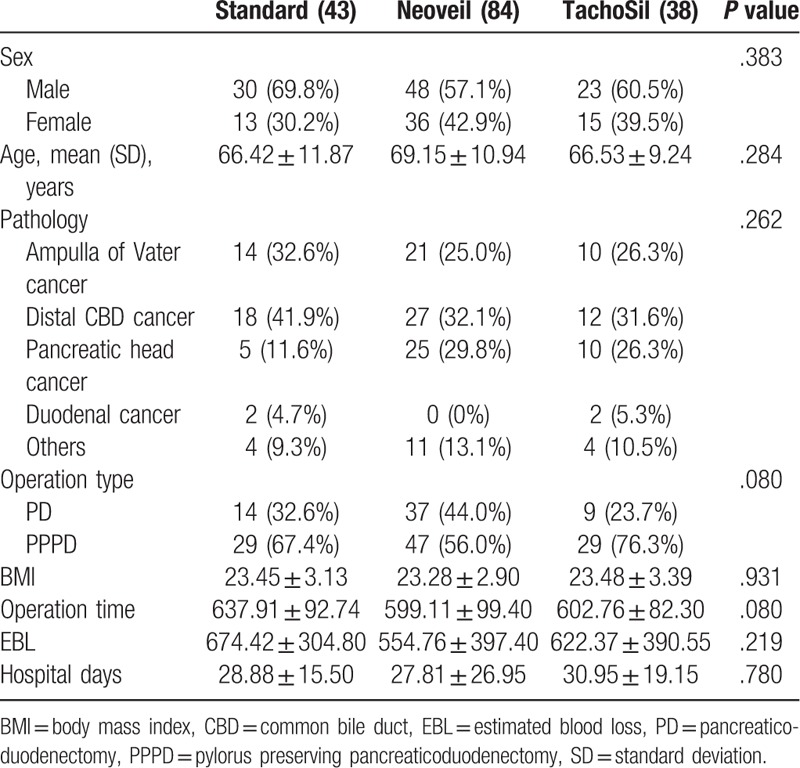
Baseline patient characteristics.

POPF grade B and C occurred in 34 patients (20.6%). These patients were more likely to be men and have a significantly longer hospital stay. The incidence of POPF grade B or above differed significantly between the groups without and with patch application (*P = *.004) (Table 2).

**Table 2:**
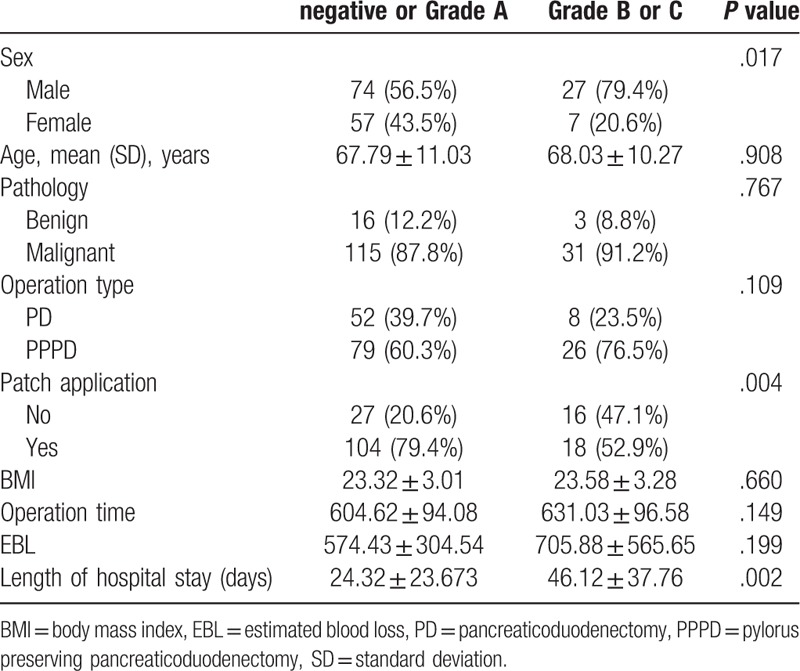
Univariate analysis of the correlation between postoperative pancreatic fistula and risk factors.

In the multivariate logistic analysis of predictive factors for POPF, male sex, no patch application, and length of hospital stay were significant independent factors of POPF grade B or above (Table 3).

**Table 3:**
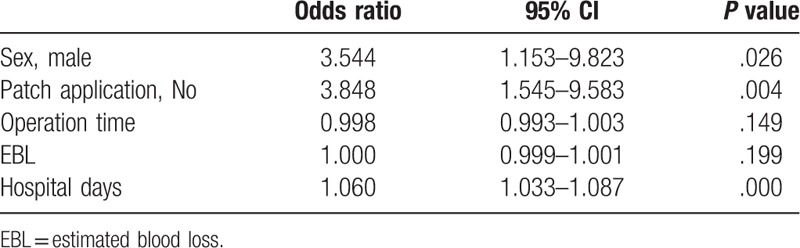
Multivariate logistic analysis of predictive factors for postoperative pancreatic fistula.

POPF grade B or above occurred in 37.2% (16/43), 14.3% (12/84), and 18.8% (6/38) of patients in the standard, Neoveil and TachoSil groups, respectively. Postoperative mortality occurred in 5 cases: 3 cases in the Neoveil group (hepatic failure, heart failure, sepsis) and 2 cases in the TachoSil group (pseudoaneurysm rupture). When comparing the standard group to either the Neoveil or TachoSil group, both the Neoveil and TachoSil groups had a significantly lower occurrence of POPF grade B or above. However, there was no significance difference between the Neoveil and TachoSil groups (Table 4).

**Table 4:**
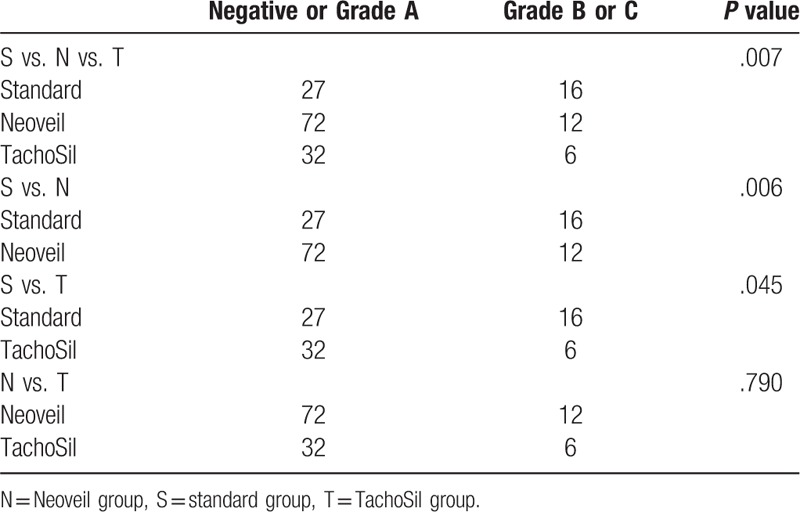
Subgroup analysis of patch application for postoperative pancreatic fistula.

Of the 165 patients, delayed gastric emptying occurred in 12 patients (7.3%), chyle leakage in 15 patients (9.0%), wound complications in 13 patients (7.9%), bile leakage in 10 patients (6.1%), gastroduodenal artery pseudoaneurysm in 14 patients (8.5%), and intra-abdominal abscess in 3 patients (1.8%). Grade IIIa or above Clavien-Dindo classification occurred in 49 patients (29.7%). When comparing the frequency of complications excluding POPF between the standard group and the patch application group, there was no significant difference (Table 5).

**Table 5:**
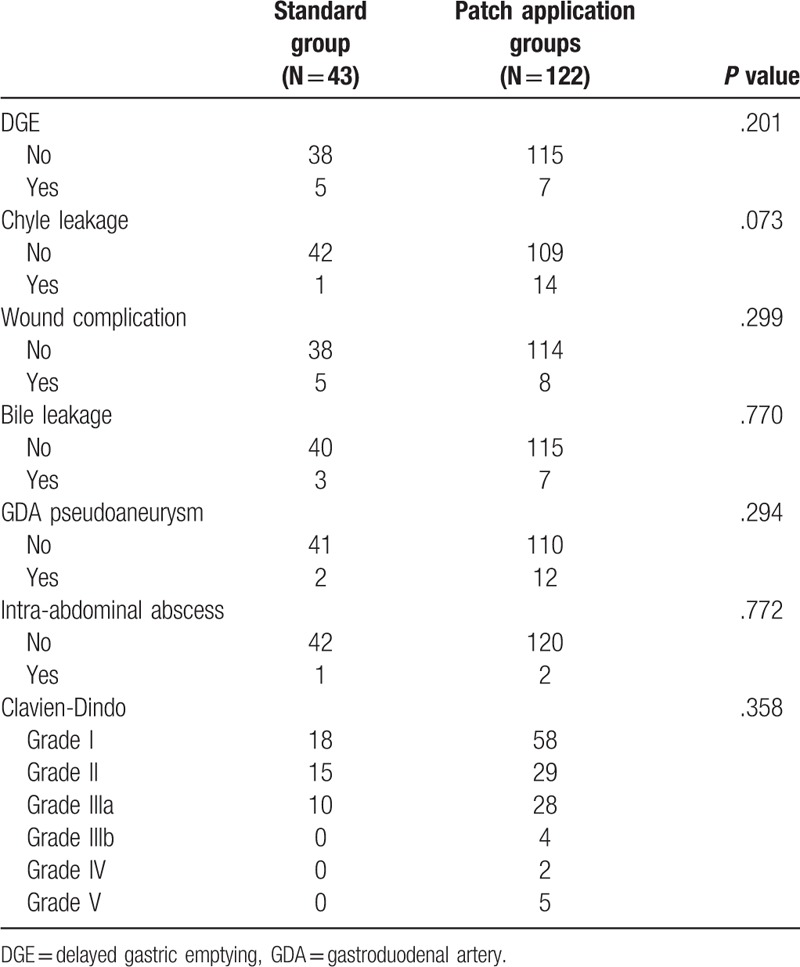
Other complications between standard and patch application groups (Neoveil and TachoSil).

## 4 Discussion

POPF is a troublesome complication after PD or PPPD. Its incidence after pancreatic surgery ranges from 5% to 40%.^[11–13]^ The mortality rate in patients with grade C POPF has been reported to be as high as 40.0%.^[14,15]^

This study was performed to investigate the efficacy of patch application with Neoveil or TachoSil in reducing POPF. The research was conducted by one surgeon who had previously performed more than 50 PD or PPPD procedures. The incidence of POPF was compared between the different patch methods according to time period. We divided the patient groups according to time period in an attempt to exclude external factors of POPF, such as pancreatic texture, pancreas duct size, and pathologic diagnosis.

POPF can lead to secondary complications such as intra-abdominal abscesses, sepsis, and bleeding. These secondary complications can give rise to prolonged hospitalization and postoperative mortality. Various techniques have been introduced to reduce the incidence of POPF including wrapping the omentum or falciform ligament^[16,17]^ and using fibrin glue to seal the pancreaticojejunostomy.^[18,19]^ In our study, there were 5 cases of mortality, 3 of which were accompanied by grade C POPF. The other 2 cases of mortality were unrelated to POPF. All mortality cases occurred in the patch application groups, but the reason is unclear. All were elderly men (ages 75–82) with heart or lung problems (dilated cardiomyopathy, coronary artery occlusive disease, or pneumoconiosis).

Neoveil is a polyethylene glycolic acid mesh, and TachoSil is a fibrinogen/thrombin-coated collagen patch that is known to strengthen tissue anastomosis and promote suturing to prevent leakage. Neoveil and TachoSil are indicated for supportive treatment in surgery, for improvement of hemostasis, to promote tissue sealing, and for suture support in vascular surgery where standard techniques are insufficient. Preliminary clinical trials have reported good results with Neoveil and TachoSil in pancreaticojejunostomy and in distal pancreatectomy.^[20–22]^

The rate of clinically significant POPF (grades B and C) was significantly reduced in the group of patients with Neoveil and TachoSil patch application compared to that in the standard group (*P* = .004). Multivariate analysis showed that male sex and patch application were correlated with the risk of pancreatic fistula. A sex correlation has not been previously reported for fistula in PD but has been noted for fistula in distal pancreatectomy.^[23]^

There was a significant decrease in the POPF seen when comparing the standard group with either the Neoveil group or the TachoSil group. However, there was no difference in the incidence of POPF between the Neoveil and TachoSil groups.

According to the largest study on the frequency of complications after PD, delayed gastric emptying was reported to range from 9% to 15%, wound infection from 8% to 10%, biliary fistula from 2% to 3%, and intra-abdominal abscess from 4% to 8%.^[24,25]^ The results in our study did not differ (delayed gastric emptying 7.3%, wound infection 7.9%, biliary fistula 6.1%, intra-abdominal abscess 1.8%), and there was no difference in the frequency of complications between the standard group and the patch application groups.

This study had several limitations, including its design as a single center, retrospective study. However, all clinicopathologic data were prospectively collected according to the same protocol, and all operations were performed by a single surgeon using the same surgical techniques, thus controlling for many possible confounding factors, including surgical and perioperative care protocols and diagnostic criteria for POPF.

This study showed that patch application with Neoveil or TachoSil may reduce the incidence of POPF after PD or PPPD.

## Author contributions

HI Seo designed and performed the experiments, analyzed the data, and wrote the manuscript; HE Kwon helped all of experiments including the hypothesis, analyzed the experimental data, and wrote the manuscript. SP Yun helped with the experiments and data interpretation.

**Conceptualization:** Hyung-Il Seo.

**Data curation:** Hye Eun Kwon, Hyung-Il Seo.

**Formal analysis:** Hyung-Il Seo, Sung Pil Yun.

**Investigation:** Hye Eun Kwon, Hyung-Il Seo, Sung Pil Yun.

**Methodology:** Hyung-Il Seo.

**Writing – original draft:** Hye Eun Kwon, Hyung-Il Seo.

**Writing – review & editing:** Hye Eun Kwon, Hyung-Il Seo.
